# Processing of harmonics in the lateral belt of macaque auditory cortex

**DOI:** 10.3389/fnins.2014.00204

**Published:** 2014-07-21

**Authors:** Yukiko Kikuchi, Barry Horwitz, Mortimer Mishkin, Josef P. Rauschecker

**Affiliations:** ^1^Department of Neuroscience, Georgetown University Medical CenterWashington, DC, USA; ^2^Brain Imaging and Modeling Section, Voice, Speech and Language Branch, National Institute on Deafness and Other Communication Disorders, National Institutes of HealthBethesda, MD, USA; ^3^Laboratory of Neuropsychology, National Institute of Mental Health, National Institutes of HealthBethesda, MD, USA

**Keywords:** communication calls, harmonics, macaques, auditory cortex, single-unit, multi-peaked neurons

## Abstract

Many speech sounds and animal vocalizations contain components, referred to as complex tones, that consist of a fundamental frequency (F0) and higher harmonics. In this study we examined single-unit activity recorded in the core (A1) and lateral belt (LB) areas of auditory cortex in two rhesus monkeys as they listened to pure tones and pitch-shifted conspecific vocalizations (“coos”). The latter consisted of complex-tone segments in which F0 was matched to a corresponding pure-tone stimulus. In both animals, neuronal latencies to pure-tone stimuli at the best frequency (BF) were ~10 to 15 ms longer in LB than in A1. This might be expected, since LB is considered to be at a hierarchically higher level than A1. On the other hand, the latency of LB responses to coos was ~10 to 20 ms shorter than to the corresponding pure-tone BF, suggesting facilitation in LB by the harmonics. This latency reduction by coos was not observed in A1, resulting in similar coo latencies in A1 and LB. Multi-peaked neurons were present in both A1 and LB; however, harmonically-related peaks were observed in LB for both early and late response components, whereas in A1 they were observed only for late components. Our results suggest that harmonic features, such as relationships between specific frequency intervals of communication calls, are processed at relatively early stages of the auditory cortical pathway, but preferentially in LB.

## Introduction

Harmonics, one of the essential acoustic structures observed in a natural environment, consist of integer multiples of a sound's fundamental frequency (F0). Natural harmonic sounds include species-specific vocalizations, a sound category with biological relevance for most species including humans (Fitch, 2006), and most musical instrument sounds. Simultaneous presentations of tonal sounds are referred to as chords; if their harmonics have a simple frequency interval ratio of 2:1 (“octave”) or 3:2 (“perfect fifth”), they are perceived as consonant by both humans and nonhuman primates (Schellenberg and Trainor, 1996; Izumi, 2000). By contrast, complex frequency interval ratios, as in a “minor second” (16:15), create roughness of sound and are perceived as dissonant. Studies have shown that human infants as young as 4 months have a preference for consonance over dissonance (Zentner and Kagan, 1996, 1998), suggesting a possible innate bias toward harmonic structure like that contained in communication sounds (but see Terhardt, 1974).

We perceive harmonically related sounds as a whole rather than as components of a spectrum. Macaque monkeys, whose architectonic structure of cortical auditory regions closely resembles that in humans (Hackett et al., 2001), judge two melodies to be the same when they are transposed by one or two octaves, but only if the melodies are tonal (Wright et al., 2000). They also perceive the pitch of harmonic sounds with a “missing fundamental” (Tomlinson, 1988), suggesting they experience gestalt perception of tonal structures just as we do.

Neurophysiological and fMRI studies of primary auditory cortex (A1) in several species have reported neurons with multiple peaks of their response rates in the frequency domain, including ones at harmonically-related intervals (monkey: Brosch et al., 1999; Kadia and Wang, 2003; cat: Oonishi and Katsuki, 1965; Sutter and Schreiner, 1991; Eggermont, 2007; Noreña et al., 2008; human: Moerel et al., 2013). Meanwhile, neuronal populations in monkey A1 show greater evoked responses to dissonant than to consonant chords (Fishman et al., 2001) and enhanced responses to mistuned harmonics compared to harmonics (Fishman and Steinschneider, 2010), perhaps due to the greater salience of the mistuned component. However, the neural basis of harmonic processing, especially outside primary auditory cortex, remains unclear.

Generally, neurons in the lateral belt (LB), located laterally adjacent to the auditory core areas, respond preferentially to complex sounds, including band-passed noise and frequency-modulated sweeps (Rauschecker et al., 1995; Rauschecker and Tian, 2004; Tian and Rauschecker, 2004). Within LB, selectivity for conspecific calls is highest in its anterolateral division (AL) (Tian et al., 2001). In the present study, the stimulus preference of auditory neurons for pure tones vs. harmonic vocalizations (“coo”) was compared in A1 and LB [including the middle lateral (ML) and anterolateral (AL) areas of auditory cortex] in behaving rhesus monkeys. We hypothesized that spectral integration of harmonically related intervals takes place preferentially in LB. Due to this integration (“spectral combination sensitivity”; Suga et al., 1979; Rauschecker et al., 1995) a sound with harmonic structure should be more effective than a pure tone, even one at the neuron's BF, in evoking a response in this region.

## Materials and methods

### Animal preparations

Two adult male rhesus monkeys (*Macaca mulatta*), weighing 7.5–11.5 kg, were prepared for chronic awake electrophysiological recording. Animal care and all procedures were conducted in accordance with the National Institutes of Health guidelines, and all experimental procedures were approved by the Georgetown University Animal Care and Use Committee. Each animal was anesthetized, and a head post and recording chamber were attached to the dorsal surface of the skull under aseptic conditions. With guidance from MRI images obtained with a 3T scanner (0.5 mm voxel size, Siemens Tim Trio), a cylindrical chamber (65° angle, 19 mm diameter, Crist Instruments, Hagerstown, MD) was positioned stereotaxically over the left hemisphere of Monkey H, and a custom-made oval chamber (20 × 40 mm, Crist Instruments) was positioned over the left hemisphere so as to cover most of the supratemporal plane of Monkey P. Monkey H had previously been used to acquire data from the rostral supratemporal plane through a rostrally positioned chamber (Kikuchi et al., 2010); therefore, for this experiment, the original chamber was removed and re-implanted over a more caudal auditory region to permit access to the middle lateral (ML) and anterolateral (AL) auditory areas in addition to the auditory core cortex. A post-operative MRI scan confirmed that the chambers were positioned correctly. The skull disc within the chamber was then removed under aseptic conditions before recording was begun. While awake, Monkey H received audiological screening, which included DPOAE (distortion product otoacoustic emission) measurements to assess cochlear function, and tympanometry to evaluate middle-ear function. The hearing ability of Monkey H was found to be normal.

### Behavioral task

Behavioral testing and recording sessions were conducted in a single-walled acoustic chamber (Industrial Acoustics Company, Bronx, NY) installed with foam isolation elements (AAP3, Acoustical Solutions). The animal sat in a monkey chair with its head fixed, facing a speaker located one meter directly in front of it in a darkened room. The animal was trained to perform an auditory discrimination task. A single positive stimulus (S+), consisting of a 300-ms pink-noise burst (PNB), was pseudo-randomly interspersed among negative stimuli (S−) for 20% of the trials. The (S−) consisted of all other stimuli. The animal initiated a trial by holding a lever for 500 ms, triggering the presentation of one of the acoustic stimuli. Lever release within a 500-ms response window after offset of the S+ led to a water reward (~0.2 ml) followed by a 500-ms inter-trial interval (ITI). Lever release in response to a negative stimulus prolonged the 500-ms ITI by 1 s (timeout). The average inter-onset-interval, including correct and incorrect trials, was 2.3 ± 0.45 s (mean ± SD). In this report, all electrophysiological analyses are based on correct trials only.

### Sound preparation

The sound waveform signals were sent through a 12-bit D/A converter (CIO-DAS1602/12, ComputerBoards) using the CORTEX dual-computer system and then amplified, attenuated, and delivered through a free-field loudspeaker (Reveal 6, Tannoy), which had a flat (±3 dB) frequency response from 63 Hz to 51 kHz.

**Figure 1:**
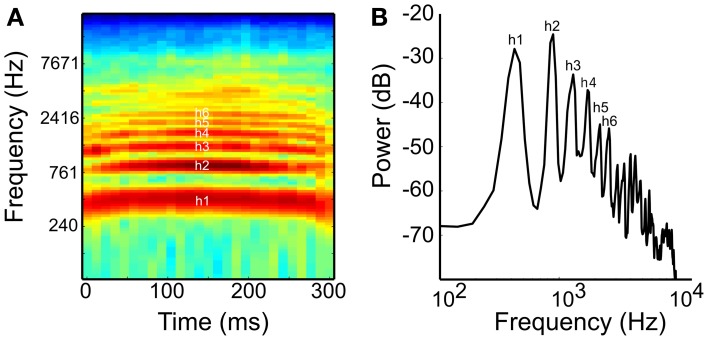
**(A)** Spectrogram and **(B)** power spectrum of the coo call used as a naturalistic harmonic sound in this study. The coo consists of the first harmonic or fundamental (h1) and five prominent harmonics with amplitudes above −50 dB (2nd to 6th harmonics, h2–h6; FFT size: 1024, Hann window). The interval between h1 and h2 is one octave and that between h2 and h3 is a perfect fifth.

All stimuli, including the monkey vocalizations (“coo” calls), had a 300-ms fixed duration, gated with a 5-ms rise/fall linear ramp. This vocalization was recorded under natural conditions in Morgan Island using a directional microphone (ME66 with K6 powering module, Sennheiser, CT, USA, frequency response at 40–20,000 Hz ± 2.5 dB) with a solid-state portable recorder (PMD670, Marantz Professional, London, UK) at a sampling rate of 48 kHz (Laboratory of Neuropsychology, NIMH). The vocalization consisted of harmonic structures with asymmetrical spectral contours (Figure 1). Pure tones (PTs) and PNBs were generated at a sampling rate of 48 kHz (32 bit) using Adobe Audition 1.5. The stimuli were normalized by recording the stimuli played through the stimulus presentation system, filtering the recorded signal on the basis of Japanese macaque audiograms (Jackson et al., 1999), and using the maximum root-mean-square (RMS) amplitude during a sliding window of 200 ms duration and presented at ~70 dB SPL. Details of the sound equalization method were described by Kuśmierek and Rauschecker (2009).

### Stimuli

The experiment consisted of several blocks of sessions, each block with a different stimulus set. After isolating a neuron, we determined the neuron's best frequency (BF) and receptive field (RF), using 23 PTs ranging from 134 Hz (C3) to 21 kHz (E10) at fifth and tritone intervals in a diatonic scale. This yielded a rough tuning curve and/or 84 PTs at semitone steps in a chromatic scale with a range of 7 octaves between 110 Hz (A2) and 13.3 kHz (G#9) to obtain a fine tuning curve. We then used a set of PTs and coos with various pitches using digital recordings of natural coo calls to test responses to complex tones (see Figure 1, Supplementary material 1). The fundamental frequency (F0) of the coo was varied using the pitch-shift function in Adobe Audition 1.5. Neural responses to PT and coo stimuli were compared using a stimulus set comprised of 10 PTs and 10 pitch-matched coo calls in either the same block (76 sessions) or in separate blocks (48 sessions). The frequency of PTs and the F0 of the coos ranged from G3 (196 Hz) to C#8 (4435 Hz) in 6 semitone steps. In each recording session, the stimuli were presented in pseudorandom order with at least 15 trials per stimulus.

### Electrophysiological recordings

Prior to each recording session, a reference point above the lateral sulcus was calculated based on the preoperative MRI scan. The position of the supratemporal plane was calculated based on the MRI images, and coordinates were mapped onto the chamber. A guide tube for up to 4 tungsten microelectrodes (0.5–3.0 MΩ, epoxylite insulation, FHC, Bowdoin, ME) was then lowered into the brain to this reference point. Each electrode was independently advanced using a remote-controlled hydraulic, 4-channel customized microstep-multidrive system (NAN-SYS-4, Plexon. Inc., Dallas, TX). As the electrode was lowered, a silent gap in the recording signal was usually observed as the electrode passed between the frontal and the temporal lobe. The post-gap depth at which the first robust spontaneous spiking activity was observed was marked as the initial recording site for each electrode; it is thus likely that much of the data were recorded from the supragranular layers. In addition to the coordinates of the recording site, which was perpendicular to the chamber grid plane, the coordinates of three additional points on the grid plane were determined as reference points. Thus, four reference points were available, as needed, to reconstruct the coordinates of each recording site in 3D space from individual anatomical MRI images (voxel size 1 mm) after each recording session. This becomes important for the standardization of the coordinates in relation to auditory areas using a population-average brain (see below for more details).

We attempted to select neurons based neither on their stimulus preference nor on the shape of their spiking activity. The signal from each electrode was passed through a head stage with gain one and high input impedance (HST/8o50-G1, Plexon Inc.) and then split to extract the spiking activity through a preamplifier system (PBX2/16sp/16fp, Plexon Inc.). The spike signals were filtered with a pass-band of 150–8000 Hz, further amplified, and then digitized at 40 kHz. Voltage-thresholding was applied to spiking activity, and spike waveforms were stored after threshold crossing. In many cases, the signal on each electrode contained activity from more than one neuron. After time-voltage thresholding, we separated the multi-unit spike trains into single-unit spike trains using the Valley Seeking algorithm (Offline Sorter, Plexon, Inc.). When we found more than one cluster, the separation quality of multi-clusters was inspected using Multivariate Analysis of Variance (MANOVA), and only data with *p* < 0.01 were considered to be separate neurons from the same electrodes and included as such in the analysis. During long recording sessions, temporal stability was sometimes lost due to electrode drift. Such instability appeared as a discontinuous cluster in time. These units were excluded from analysis.

We also inspected the inter-spike interval (ISI) for each cluster. An ISI distribution with entries smaller than the refractory period (1 ms) signifies that the recorded spikes were from more than a single neuron. In most such cases, we changed the threshold at the stage of voltage-thresholding and re-ran the cluster analysis. However, if an ISI < 1 ms was still observed in a small proportion of a newly sorted ISI distribution (usually <0.3%), the furthest spike waveform from the cluster center in 2D feature space with ISIs less than the refractory ISI was removed from the unit.

Time stamps indicating the timing of auditory stimulus, behavioral response, and reward events were sent through CORTEX (CIO-DAS1602/12, CIO-DIO24, ComputerBoards), and continuous data, such as sound waveforms and eye movements monitored by an infrared-based eye-tracking system at 60 Hz (ETL-200, ISCAN, Inc.), used to check the animals' state of wakefulness, were sent to a Multichannel Acquisition Processor system (MAP, Plexon, Inc.) and then integrated with the spike data. During the recording session, spikes were roughly sorted by real-time acquisition programs using template matching and PCA clustering methods (RASPUTIN, Plexon), and rough estimations of the frequency- and intensity- tuning profiles of the neuron were examined online (Neuroexplorer, Nex Technologies, MA). Throughout the recording sessions, we monitored neuronal activity visually with an oscilloscope (HM407-2, HAMEG) and aurally through headphones (HD 280 Professional, Sennheiser). Data selection, pre-processing, and data analysis were performed using MATLAB and SPSS. All the results in this report are based on offline analysis conducted after the experiments were completed; the online analysis was used only as a quick evaluation of a neuron's characteristics for stimulus selection purposes.

### Data analysis

The spike trains of single-unit activity (SUA) were binned at 1 ms for each trial and the average spontaneous firing rate and its variability per stimulus condition was first calculated during the baseline period (0–150 ms before sound onset). The spike trains were convolved with a Gaussian kernel (σ = 10) to construct spike-density peri-stimulus time histograms (PSTHs) and then normalized to the average variability (SD) of the raw baseline firing rate across all stimulus conditions. Neurons that showed responses 2.0 SDs above baseline for 10 consecutive 1-ms sampling points in the normalized PSTH to at least one sound (other than the S+, pink noise) were defined as “auditory-responsive.” These constraints were imposed in order to exclude spurious activity or artifacts.

Tuning curves were constructed based on peak response magnitude (i.e., the maximum magnitude of the peak firing rate minus the average baseline firing rate) and then smoothed by moving the average along with the two neighboring points (i.e., two semitones) on each side of the frequency axis. The frequency that produced the maximum response on the tuning curve function was defined as the best frequency (BF) for the neuron. Neurons were classified as having either one peak on the frequency tuning curve function (i.e., a single-peaked neuron) or more than one peak (i.e., a multi-peaked neuron) with a clear excitation greater than 2.5 SDs above baseline firing rates and the half driven rate of the peak (i.e., 50% firing rates of the normalized peak magnitude in response to the BF). To obtain a clear tuning peak, we used this stricter criterion than the one defined above for auditory-responsive neurons.

Neuronal latency was calculated based on the spike density function with the Gaussian kernel (σ = 10) described above and was defined as the time from sound onset to the first millisecond bin in which spiking activity rose 2 SDs above baseline for 10 consecutive 1-ms bins. The SD calculated from the raw data (taking the grand average of variability across all stimulus conditions) generally yielded a higher value than that calculated from smoothed data. Minimum latency was defined as the shortest latency across all auditory responses of the neuron; this was sometimes different from the latency in response to the BF, which was measured from stimulus onset to the peak magnitude of the response. Minimum latency to S+ was calculated only when the neuron showed a significant response to S+ in both correct and incorrect trials. For incorrect trials, the recording session was included in the statistical analysis only if there were at least five “miss” trials (without a response to S+) within that session. If there were other types of errors (e.g., premature response to the positive stimulus), the trials were excluded altogether to avoid incorporating artifactual effects on neuronal activity, e.g., effects of motor responses. BF latency was defined as the latency in response to the BF, and the average latency was defined as the median latency across all auditory responses of the neuron. The coo-call latency was defined as the latency in response to the coo whose F0 was matched to the neuron's BF. Neuronal latencies and spike rates across subdivisions of auditory cortex were compared using the Kruskal-Wallis test, and *post-hoc* testing between subfields was performed using Tukey's “honestly significant difference” (HSD) test to correct for multiple comparisons.

To analyze the tuning width of each neuron quantitatively, a bandwidth index (BI) was calculated by a method similar to one used by Lakatos et al. (2005; formula shown in **Figure 4A**) using a fine-tuning paradigm. The BI was calculated using normalized firing rates during the entire sound duration (0–300 ms) after subtracting mean baseline firing rates across all stimuli in the single-peaked neurons (**Figure 4A**). A BI index close to 1 indicates sharp frequency tuning, whereas a BI index near 0 indicates broad tuning. We also measured the traditional tuning width of the neuron's response peak at 30 dB above threshold (BW30; Sutter and Schreiner, 1991; Schreiner and Sutter, 1992). Pure tones with sound durations of 300 ms at five different intensities in 10-dB steps (30–80 dB SPL) were presented at different frequencies (E3–E10, 165-21 kHz) in octave steps. The tones were played in a pseudorandom order of different frequencies and intensities. The neuron's frequency response area (FRA) was determined as a contour line of 2.5 SD above baseline activity in the frequency and intensity domains, and the tuning width at an intensity of 30 dB SPL above the neuron's threshold in the FRA was determined as the neuron's BW30. To make the bandwidth results directly comparable between our study and Sutter and Schreiner's study, we computed the BW 30 using both single- and multi-peaked neurons. Since we used a fixed stimulus set (40 tones), we were not always able to precisely determine the neuron's threshold, because some neurons still showed a response at the lowest sound intensity we employed. In this case we calculated the BW30 as the tuning width at 60 dB SPL, which is 30 dB above the lowest sound intensity we used. For the same reasons, if the neuron's threshold was as high as 60 dB SPL, we were not able to obtain the BW30. Also, due to time constraints, we were not always able to fully determine the neuron's FRA after completing the other tests; thus our analysis for BW30 is limited to the neurons we actually tested in this paradigm.

Multi-peaked neurons were tested with a fine-tuning paradigm that included 84 pure tones in chromatic scales (A2-G#9, 110–13289.8 Hz). We selected the best two (if there were only two) or three peaks (>2.5 SDs above baseline and the half-driven rate of the peak) and assigned them to BF1–BF3 in ascending order of their frequency at the peaks (i.e., the lowest peak frequency was assigned to BF1). Among these frequencies, the frequency that elicited the greatest peak response was selected as the neuron's overall BF. The criterion for the presence of a peak was a response above a specific threshold; the criterion for two peaks was a decrease in response below this threshold for at least one point between the two peaks, the minimum inter-peak interval being separation by more than two semitones. The frequency interval ratio (BF ratio) was calculated for all three combinations (BF1–BF2, BF1–BF3, and BF2–BF3) and normalized by the frequency of the lowest peak, a method similar to that used by Kadia and Wang (2003). The distribution of BF ratios was then binned by one tenth of an octave (Sutter and Schreiner, 1991), which is wider than semitone resolution. The distribution of BF ratios was calculated based on the peak firing rates during the early-response period (0–70 ms from sound onset) and during the late-response period (71–300 ms from sound onset). The 70-ms time window was used to separate the onset and sustained components of the response, since a typical auditory single-unit response showed a trough between onset and sustained responses at approximately 60–80 ms. The confidence interval (CI) was calculated from the distribution of BF ratios using the same bin width. We also calculated the CI from the distribution of BF ratios under the assumption that the peak interval relations of multi-peaked neurons were random. The number of occurrences of peak intervals was assigned to a given bin of BF ratio and the averaged distribution after 1000 permutations was computed. Since the CI from the average distribution was always lower than the former CI using the raw distribution, we employed the CI calculated from the raw distribution in this study. To compare the number of harmonic intervals in multi-peaked neurons, we used one or two bins that were centered at the perfect fifth (1.5) and octave (2.0). When the BF ratio was in the middle of two bins, we used the bin with maximum peak.

Data from the subfields of LB (i.e., ML and AL) were grouped whenever the sample size for individual subfields was too small to allow for statistical testing.

### Assignment of recording locations to cortical areas

The recording sites in this study were assigned to either the auditory core region (primary auditory cortex, A1) or to the auditory LB region [middle lateral field (ML) and anterolateral field (AL)] using the following criteria.

**Figure 2:**
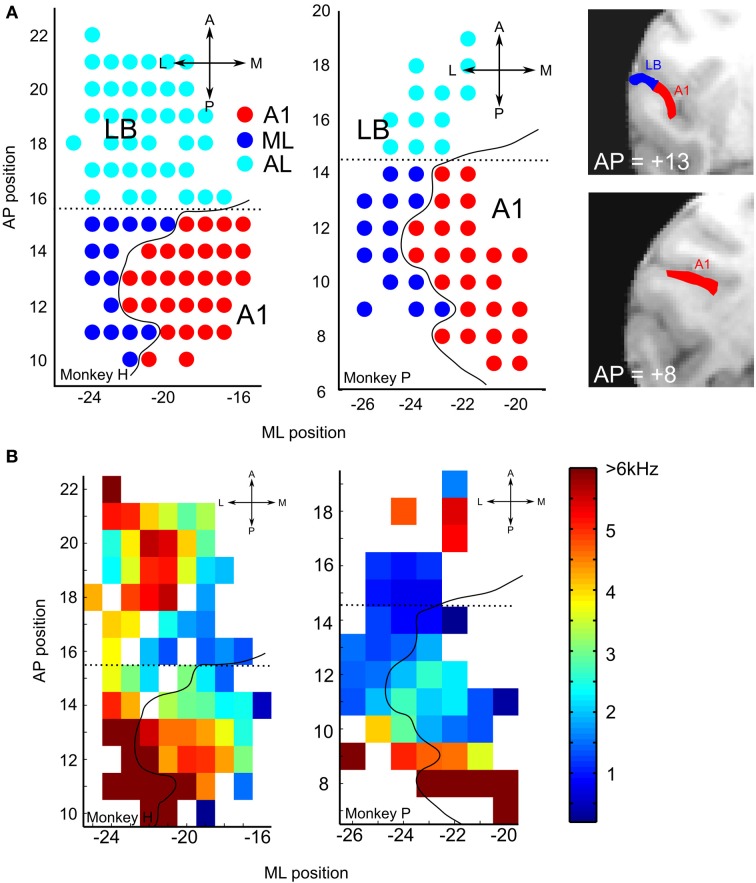
**(A)** Recording sites: the location of A1 and lateral belt (LB) are depicted on the left supratemporal plane (STP) of Monkeys H and P. The anterior-posterior (AP) and medial-lateral (ML) coordinates were transformed into standardized coordinates based on the population-average macaque brain (McLaren et al., 2009, 2010). The curved solid black line on each map shows the estimated border between core and LB based on the atlas from a single subject (Saleem and Logothetis, 2007). The anterior-posterior border (dotted line) was drawn from the frequency reversal observed on mapping the best frequencies (BFs, see Figure 2B); this reversal occurred at a slightly different AP coordinate in the two monkeys (Monkey H, 15.5; Monkey P, 14.5). Shown on the right are two coronal MRI images of monkey P at the indicated AP levels, with A1 and LB on the supratemporal plane (STP) highlighted in red and blue, respectively. **(B)** Best frequency (BF) maps for each of the two animals. Frequency reversal on the BF maps was used to determine the anterior-posterior boundary of the auditory subdivisions for each animal. The dotted line was calculated based on the lowest frequency reversal point using mean values, smoothed by a 3-mm sliding window, along the AP direction. The scale bar on the right shows the frequency range of pure tones used to estimate the neurons' BF.

To reconstruct the boundaries between cortical areas along the anterior-posterior (AP) axis, in particular the boundary between ML and AL, we employed the standard approach of using the cortical tonotopic gradient map based on the neurons' best frequency (BF, Figure 2B; cf. Rauschecker et al., 1995). The mean BF along the AP axis was used to calculate the reversal point of the BF tuning curve along the AP axis (monkey H, 15.5; monkey P, 14.5, Figure 2A).

To reconstruct the boundaries between cortical areas along the medial-lateral (M-L) axis, i.e., between the putative core and LB regions, a similarly precise approach based on functional criteria cannot be taken, even though the neurons' response characteristics, in particular bandwidth tuning or tone-vs.-bandpass-noise preference, do differ between core and LB (Rauschecker et al., 1995). Therefore, we used an approach based on population-average T1-weighted MRI images (112RM-SL) provided by McLaren et al. (2009, 2010; the standardized atlas of the rhesus macaque brain can be downloaded from: www.brainmap.wisc.edu/monkey.html). This approach permits us to standardize electrophysiologically identified regions into a common space across multiple individuals and to assign the xyz-coordinate of each recording site to one of these regions. Most importantly for our study, these coordinates can be used to identify the medial-lateral boundary between core and LB.

The 112RM-SL database is the average of 112 rhesus macaque brains co-registered with the single-subject atlas (D99-SL) of Saleem and Logothetis (2007), which was itself co-registered with histological slices (Nissl, parvalbumin, SMI-32, calbindin and calretinin) aligned to cytoarchitectonic areas. We used the Analysis of Functional NeuroImages (AFNI) software (Cox, 1996) for MRI processing. The volumes were reconstructed from the original T1-weighted image of an individual animal in AFNI using the *to3d* function. To mask out nonbrain areas occupied by a thick mass of muscle around the skull, the skull was removed from the images using the *3sSkullStrip* function, and the skull-stripped images were then aligned to the population-average brain (112RM-SL) to generate a transformation matrix that converted each xyz-coordinate of the recording site into standardized space. Since inter-individual variability is large, especially across the width of the supratemporal plane (STP), each co-registered brain yielded discrepancies for some brain structures, especially when the single-subject atlas was the reference for each site. In that case, we chose the gray matter closest to the site and visually assigned the cortical region based on the atlas.

## Results

The animals performed an auditory discrimination task on average at 96.3% accuracy (86.4% correct responses for the S+ trials and 98.8 % correct for the S− trials). Most errors (1.8%) were failures to release the lever to the S+ (“miss” errors); the other types of errors were either premature responses to the S+ [lever release before sound offset (0.9%)] or “false-alarm” errors [lever release to a negative stimulus (0.9%)].

Neurons in A1 and LB were recorded either separately or, more often, simultaneously using two to three electrodes. The spontaneous firing rates showed no significant differences across the three divisions of auditory cortex (monkey H: A1, 11.7 ± 8.6 spikes/s; ML: 15.5 ± 10.9 spikes/s; AL: 11.2 ± 8.1 spikes/s, *p* = 0.07; monkey P: A1, 14.0 ± 10.7 spikes/s; ML: 16.9 ± 12.4 spikes/s; AL: 15.0 spikes/s ± 9.8 spikes/s, *p* = 0.18, Kruskal-Wallis test, mean ± SD).

### Hierarchical processing in three subdivisions of auditory cortex (A1, ML, and AL) in response to pure tones (PT) and pink-noise bursts (PNB)

**Figure 3:**
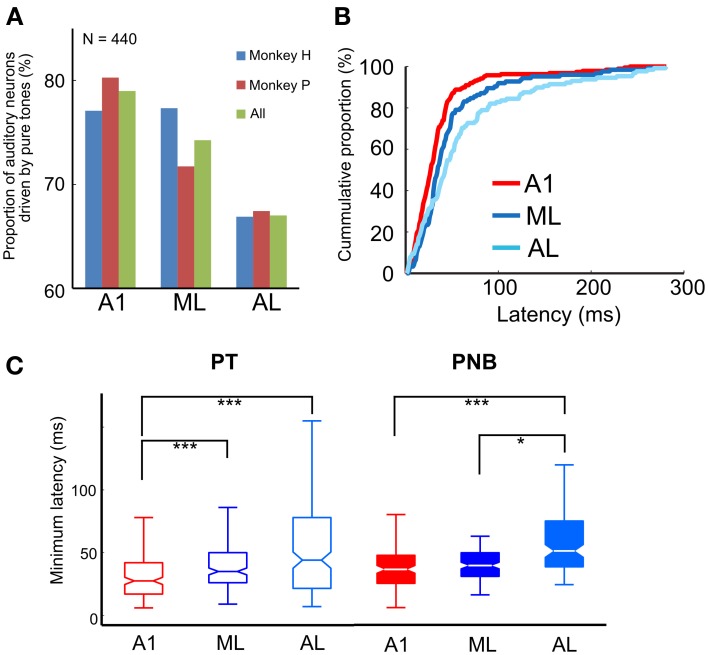
**(A)** Proportion of auditory neurons driven by pure tones in A1, ML, and AL. The proportion was greatest in A1, followed by ML and AL. **(B)** Cumulative proportion of minimum response latency to pure tones. Latencies in LB (i.e., ML and AL) were significantly longer than in A1 [median: A1: 28 ms, *N* = 188; ML: 35 ms, *N* = 124; AL: 44 ms, *N* = 128; A1 vs. ML, *p* < 0.001; A1 vs. AL, *p* < 0.001 (Tukey's HSD test)]. **(C)** Minimum response latency to pure tones (PT) (left) and pink-noise bursts (PNB) (right) across the three subdivisions of auditory cortex. The central marks of the boxplots show the median latency between the 25th and 75th percentiles. The asterisks denote the significance level of *post-hoc* testing (Tukey's HSD test, ^*^p < 0.05, ^***^p < 0.001).

We first analyzed the responses of 596 single neurons (A1, 238; ML, 167; AL, 191) to pure tones (PTs). All three subfields of the auditory cortex generally responded to the PTs across a wide range of frequencies. The proportion of auditory neurons that showed a significant response to PTs decreased gradually from A1 to ML to AL (A1, 79%; ML, 74%; AL, 67%; see Table 1 and Figure 3A). Although there was no overall statistically significant effect of PT responsiveness across subdivisions (chi-square test, χ^2^ = 2.1, *df* = 2, *p* > 0.05), minimum onset latencies to PTs, i.e., the shortest latencies among all the responses of a neuron, differed significantly across the three subdivisions (*p* < 0.001, Kruskal-Wallis test), being shortest in A1 [median: 28 ms, 25th percentile (*Q*_1_) = 17 ms, 75th percentile (*Q*_3_) = 42 ms, *N* = 188] followed by ML and AL (ML: median: 35 ms, *Q*1 = 26 ms, *Q3 = 50* ms, *N* = 124, *p* < 0.001; AL: median: 44 ms, *Q1 = 22 ms*, *Q*_3_ = 78 ms, *N* = 128, *p* < 0.001, Tukey's HSD test, Figure 3B, Table 2). Minimum latencies to the PNBs on correct trials were also compared across the three subdivisions: Like the gradual change in minimum latency observed in response to the PTs (Figure 3C, left), the latency to the PNBs differed significantly across the subdivisions (Figure 3C, right, *p* < 0.001, Kruskal-Wallis test). The *post-hoc* tests show that the median latency in A1 and ML differed significantly from that in AL, though the A1 and ML latencies did not differ from each other (A1: median: 36 ms, *Q*1 = 25 ms, *Q*3 = 47 ms; ML: median: 39 ms, *Q1 = 31* ms, *Q*3 = 49 ms; AL, median: 51 ms, *Q*1 = 38 ms, *Q3 = 74* ms, A1 vs. AL: *p* < 0.001; ML vs. AL: *p* < 0.05, Tukey's HSD test). To understand the variability between the two monkeys and three subdivisions of the auditory cortex, “monkey” and “area” were included as between-subject condition factors in Two-Way ANOVAs. The analysis revealed that for PT minimum latencies, there was a significant main effect of area [*F*_(2, 434)_ = 4.929, *p* < 0.01] and monkey [*F*_(1, 434)_ = 12.134, *p* < 0.01] but no interaction [*F*_(2, 434)_ = 1.867, *p* = 0.16]. For PNB latencies, there was a significant main effect of area [*F*_(2, 209)_ = 4.375, *p* < 0.02] but not monkey [*F*_(1, 209)_ = 0.3495, *p* = 0.56] and there was no interaction [*F*_(2, 209)_ = 0.14, *p* = 0.87]. The main effect of area was present for both PT and PNB latencies. Together, these data suggest that sound processing occurs along a cortical hierarchy from A1 to ML and AL.

**Table 1:** **Population of auditory neurons driven by pure tones (PT) in different subfields of auditory cortex**.

**Subject**	**A1 %**	**ML %**	**AL %**	**Total**
Monkey H	74/96 (77)	58/75 (77)	99/148 (67)	231/319
Monkey P	114/142 (80)	66/92 (72)	29/43 (67)	209/277
Total	188/238 (79)	124/167 (74)	128/191 (67)	440/596

**Table 2:** **Minimum latencies to pure tones (PT) and pink noise bursts (PNB) in different subfields of auditory cortex**.

**Stimulus type**	**Subject**		**A1**	**ML**	**AL**	**Tukey's HSD test**		
						**A1 vs. ML**	**A1 vs. AL**	**ML vs. AL**
PT	Monkey H	Q1 (25%)	17	28	23	*p* < 0.01	*p* < 0.001	NS
		Q2 (50%)	30	40	45			
		Q3 (75%)	42	61	91			
		N	74	58	99			
	Monkey P	Q1 (25%)	16	19	19	*p* < 0.05	NS	NS
		Q2 (50%)	27	31	41			
		Q3 (75%)	38	45	57			
		N	114	66	29			
	Total	Q1 (25%)	17	26	22	*p* < 0.001	*p* < 0.001	NS
		Q2 (50%)	28	35	44			
		Q3 (75%)	42	50	78			
		N	188	124	128			
PNB	Monkey H	Q1 (25%)	25	41	39	NS	*p* < 0.001	NS
		Q2 (50%)	32	42	53			
		Q3 (75%)	42	46	74			
		N	52	4	29			
	Monkey P	Q1 (25%)	26	29	37	*p* < 0.05	NS	NS
		Q2 (50%)	37	39	43			
		Q3 (75%)	48	50	73			
		N	72	45	13			
	Total	Q1 (25%)	25	31	38	NS	*p* < 0.001	*p* < 0.05
		Q2 (50%)	36	39	51			
		Q3 (75%)	47	49	74			
		N	124	49	42			

We also compared the electrophysiological responses to the S+ (PNB) during correct trials (17.3% of all trials) and incorrect (“miss”) trials (1.8% of all trials; see Methods). The minimum response latency for correct and incorrect trials during the same recording session did not differ significantly in either A1 or LB (A1: median: 35 vs. 37 ms, Q1:22 vs. 21 ms; Q3:47 vs. 47 ms, respectively, *N* = 54, *p* = 0.20; LB: median: 46 vs. 45 ms, Q1: 34 vs. 33 ms, Q3: 62 vs. 66 ms, respectively, *N* = 40, *p* = 0.41, Wilcoxon signed-rank test). The latencies were generally longer in LB than in A1 for both trial types (correct trials, *p* < 0.001; incorrect trials: *p* < 0.05), as expected from the responses to PTs and PNBs, consistent with the notion of a cortical hierarchy from A1 to LB.

### Multi-peaked neurons with harmonically related intervals in A1 and LB

We constructed a frequency tuning curve using the neuron's peak magnitude to calculate the best frequency (BF) of each auditory neuron. Among 205 neurons recorded in the fine-tuning pure-tone paradigms using chromatic scales with semitone steps, 142 neurons (69%) were single-peaked, and 63 neurons (31%) were multi-peaked. The proportion of multipeaked neurons in A1 and LB did not differ significantly in Monkey P (A1 vs. LB: 47 vs. 37%, A1: *N* = 17; LB: *N* = 10; χ^2^ = 0.65, *df* = 1, *p* > 0.05) but it decreased in Monkey H (35 vs. 17%, A1: *N* = 16; LB: *N* = 16; χ^2^ = 5.85, *df* = 1, *p* < 0.05). The distribution of BFs for multi-peaked neurons (*N* = 63) was not significantly different from that for single-peaked neurons (*N* = 142), when the BFs eliciting the neurons' maximum peak response were compared (Monkey H: mean ± SE, 2826 ± 619 Hz vs. 3625 ± 430 Hz, *p* = 0.63; Monkey P: 3244 ± 779 Hz vs. 2560 ± 614 Hz, *p* = 0.28, multi-peaked vs. single-peaked, Wilcoxon rank-sum test).

**Figure 4:**
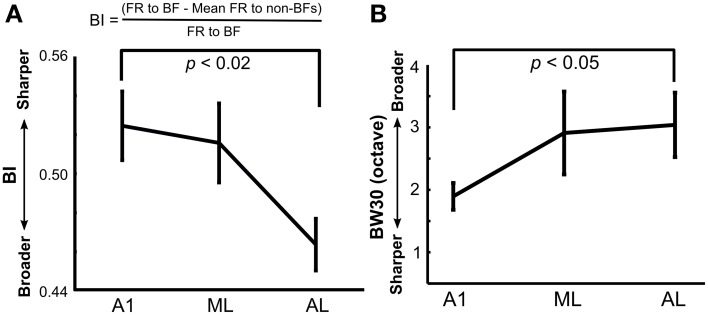
**Tuning width in A1, ML, and AL measured using **(A)** a bandwidth index (BI) and **(B)** a traditional BW30 index**. BI was calculated based on the formula shown using the fine-tuning paradigm (see Methods). There was a main effect of recording site on BI (Kruskal-Wallis test: *p* < 0.02) with significantly sharper tuning in A1 compared to that in AL (Tukey's HSD test, *p* < 0.01). Tuning width using BW30 gave similar results with significantly sharper tuning in A1 than AL (*p* < 0.05, Wilcoxon rank sum test). Means are plotted and standard errors are represented by bars.

We next analyzed the sharpness of frequency tuning in A1 and LB using a bandwidth index (BI; see Methods) similar to the one used by Lakatos et al. (2005), with a BI close to 1 indicating sharp frequency tuning, and a BI near 0 indicating broad tuning. There was a main effect of recording site on BI (A1: 0.52 ± 0.02, *N* = 47; ML: 0.52 ± 0.02, *N* = 36; AL: 0.46 ± 0.01, *N* = 59 (mean ± SE), Kruskal-Wallis test, *p* < 0.02, Figure 4A) with A1 neurons displaying sharper tuning compared to AL (Tukey's HSD test, *p* < 0.02). Tuning width was further examined in a subset of neurons with the traditional approach measuring BW30 (Sutter and Schreiner, 1991; see Methods). This analysis had a similar outcome with a significant difference in frequency tuning width between A1 and AL (A1: 1.9 ± 0.21 octaves, *N* = 29; ML: 2.9 ± 0.66 octaves, *N* = 19; AL: 3.0 ± 0.51 octaves, *N* = 14, mean ± SE, *p* < 0.05, Wilcoxon rank sum test, Figure 4B).

**Figure 5:**
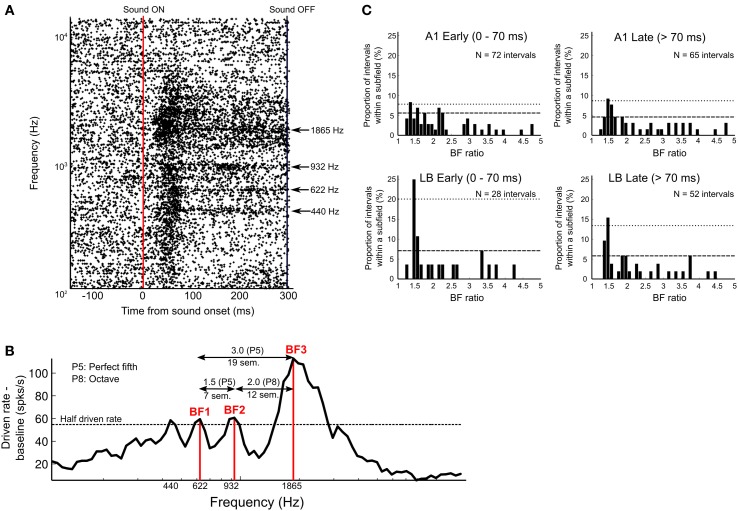
**Response of multi-peaked neurons. (A)** Raster plots of single-unit activity in an example neuron from area ML with multi-peaked tuning (bin width 1 ms). Plots are aligned to sound onset (vertical red line) in response to 84 tones presented in semitone-steps between 110 Hz (A2) and 13.3 kHz (G#9). Sound offset is indicated by vertical black line at far right. Y-axis indicates frequency (Hz) of pure-tone stimuli on a log scale. The neuron responded to four distinct frequency bands (440, 622, 932, and 1865 Hz), which are indicated by arrows. **(B)** Rate tuning curve of the multi-peaked neuron shown in **(A)** based on peak response magnitude during the entire duration of the sound (normalized by subtracting baseline activity and 5-point smoothing). Four peaks above the half driven rate (defined as 50% of the highest normalized peak firing rate, here indicated as a dotted line) were detected, and we chose the three highest peaks as best frequencies (BF1, BF2, BF3) to analyze the interval relations of multiple peaks. In this neuron, the frequency interval ratios of the three peaks were 7, 12, and 19 semitones, which correspond to 1.5 (BF2/BF1), 2.0 (BF3/BF2), and 3.0 (BF3/BF1), all of which are harmonically related (i.e., perfect harmonic or musical intervals): P5 (perfect fifth), 7 semitones apart; P8 (perfect eighth, or octave), 12 semitones apart, and another P5 (perfect fifth), 19 semitones apart. **(C)** Distribution of peak distance in multi-peaked neurons of A1 and LB. The distribution of BF ratios was calculated based on the peak firing rates during the early-response period (0–70 ms from sound onset) and during the late-response period (71–300 ms from sound onset). The interval distance between two peaks was estimated based on frequency interval ratio (BF ratio, x-axis), and the relative frequency (i.e., number of intervals relative to the total number of intervals in each subdivision) is shown on the y-axis. The confidence interval (CI) at 99.5% is indicated by a dashed line, and at 99.9%, it is indicated by a dotted line. BF ratios above 5.0 are not shown in the figure for display purposes; however, none of those peaks reached the CI threshold of 99.5%.

Figures 5A,B show an example of a single neuron from area ML with a multi-peaked response. Whereas the tuning of the excitatory onset response was broad across a wide range of frequencies, several discrete frequency peaks can be distinguished in the sustained response after the drop-off of the initial onset response (see raster plots in Figure 5A). This neuron's BF (frequency with highest peak response; 112.8 spikes/s) was 1865 Hz (A#6). However, the sustained response showed three additional peaks above threshold, which were all distinct in frequency (440 Hz = A4, 622 Hz = D#5, and 932 Hz = A#5). The best three peaks were chosen based on the peak firing rates and assigned as BF1 (D#5, 59.4 spikes/s), BF2 (A#5, 60.6 spikes/s), and BF3 (A#6, 112.8 spikes/s) in order of ascending frequency (see Methods). The frequency ratios of the best three BFs in relation to each other were 3.0 (BF3/BF1, 19 semitones), 1.5 (BF2/BF1, 7 semitones), and 2.0 (BF3/BF2, 12 semitones), which correspond to “perfect” harmonic or musical intervals (i.e., ratios of 2.0 = octave; and ratios of 1.5 and 3.0 = “perfect fifths”).

If LB contributes more than A1 to the spectral integration of harmonically-related interval information, the distribution of distances between two peaks of multi-peaked neurons might tend toward harmonically-related interval ratios more often in LB than in A1. We calculated the interval ratio between best frequencies (BF ratio) in all multi-peaked neurons (Figure 5C). This was done separately for early (0–70 ms from sound onset) and late responses (>70 ms). The distribution of BF ratios in LB showed a maximum at the perfect-fifth interval (3:2 = 1.5) in both the early and late periods (above the confidence interval (CI) at 99.9%) and at the octave (2:1 = 2.0, above the CI at 99.5%) for late periods, whereas BF ratios in A1 showed a peak at the perfect-fifth interval only in the distribution of late responses (above the CI at 99.9%). A significant difference in the distribution of peak distances was found between A1 and LB for early (A1: *N* = 72 intervals measured, LB: *N* = 28, *p* < 0.01, Wilcoxon signed rank test) but not for late responses, when the same bin-by-bin paired comparison was performed (A1: *N* = 65 intervals, LB: *N* = 52 intervals, *p* = 0.60). The proportion of harmonic intervals in the early period was significantly greater in LB than in A1, and the different bin widths did not affect the results (bin width = 2: 39 vs. 15%, χ^2^ = 5.28, *df* = 1, *p* < 0.025; bin width = 1, 25 vs. 4%, χ^2^ = 8.75, *p* < 0.005, χ^2^ test).

### Response to pitch-shifted coos

If, as hypothesized, the spectral integration of harmonically related frequencies takes place in LB, a sound with harmonic structure should be more effective in evoking a response in this area than would a pure tone, even one at the BF. To test this hypothesis, we shifted the pitch of a coo call to match the neuron's BF and compared the responses between A1 and LB. A coo call was used because a previous study showed that LB neurons can be driven quite selectively by species-specific vocalizations (Tian et al., 2001). Auditory responses were sometimes elicited by a coo with the same pitch as a low tone sharing the same F0, particularly in neurons responsive to low frequencies, even if the coo's overtones were outside the neuron's excitatory receptive field (RF).

**Figure 6:**
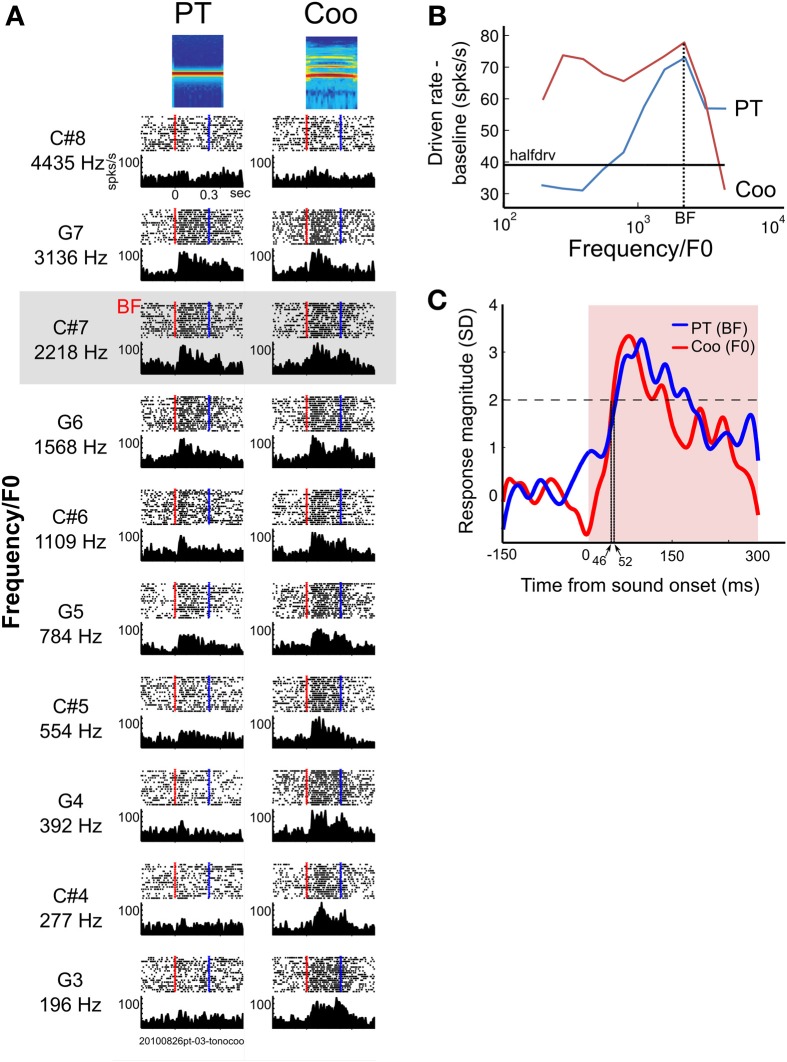
**Example of a neuron in A1 showing frequency-representative responses to pure tones and pitch-shifted coos**. **(A)** Spike rasters (upper half of each graph) and PSTHs (lower half) aligned to sound onset (red dotted line) in response to pure tones (left column) and pitch-shifted coos (right column) at the same pitch (196–4435 Hz at half-octave steps). The blue line marks sound offset. **(B)** Tuning curves for Unit A, shown in **(A)**. The tuning curves resulting from peak responses to PTs and to the pitch-shifted coo, which is based on the fundamental frequency (F0) of the coos. Black horizontal line indicates half-driven rate. This type of neuron continued to show high firing rates when the overtone harmonics of the pitch-shifted coo fell into the neuron's RF, even when the F0 of the coo was outside the RF. **(C)** Averaged PSTH of the responses of Unit A to its BF (solid blue line) and to a coo (solid red line) whose F0 was matched to the BF. The latency in response to the BF-PT was 46 ms, whereas the latency in response to the coo was 52 ms. Sound duration period is shown in pink; sound onset is indicated by the vertical edge at time zero.

The classification of neurons was based on each neuron's RF, and this was limited to neurons (*N* = 24) whose lower PT frequency cutoff fell within the range of frequencies we used in this paradigm (196–4435 Hz, see Methods). The neurons were classified into two groups: (1) frequency-representative neurons that responded to coo stimuli when the overtone harmonics fell into the neuron's RF, even though the F0 of the coo was outside the RF; (2) pitch-selective neurons that responded when the F0 of coo stimuli fell into the RF but did not respond to coo stimuli when the overtone harmonics fell into the neuron's RF. Other types of neurons showed various kinds of responses that deviated from the above two groups; these neurons were categorized as “non-classified” (*n* = 64). Figure 6A illustrates an example of a neuron in A1 that showed a frequency-representative response (Unit A). This neuron had a single peak (Figure 6B), and its BF was 2218 Hz (C#7). Since the overtone harmonics (h2–h6, Figure 1) fell into the neuron's RF even when the F0 of the coo was outside the RF, the tuning curve in response to pitch-shifted coo calls was broader than that in response to PTs (Figure 6B). The onset latency to the BF was 52 ms, whereas the latency in response to the coo (whose F0 matched the BF) was 46 ms, with no difference in mean firing rate to the two stimuli (PT, 62.2 ± 20.7 spikes/s; coo, 73.2 ± 19.9 spikes/s; *p* = 0.13, Wilcoxon ranksum test, Figure 6C). Of the 88 neurons tested, 21 (24%) were of this type, and there was no difference in proportion between A1 and LB (8 vs. 13 neurons, respectively, χ^2^ = 2.1, *df* = 1, *p* = 0.15). Although the number is small (*N* = 3), there were neurons that exhibited similar tuning in response to PTs and pitch-shifted coos with a shorter latency to the coo than to the PT-BF, and an enhanced response to the coo relative to the response to the PT-BF (Supplementary Figure 1).

### Average response latencies to pure tones at the BF and to F0-matched complex tones (“coo” calls)

**Figure 7:**
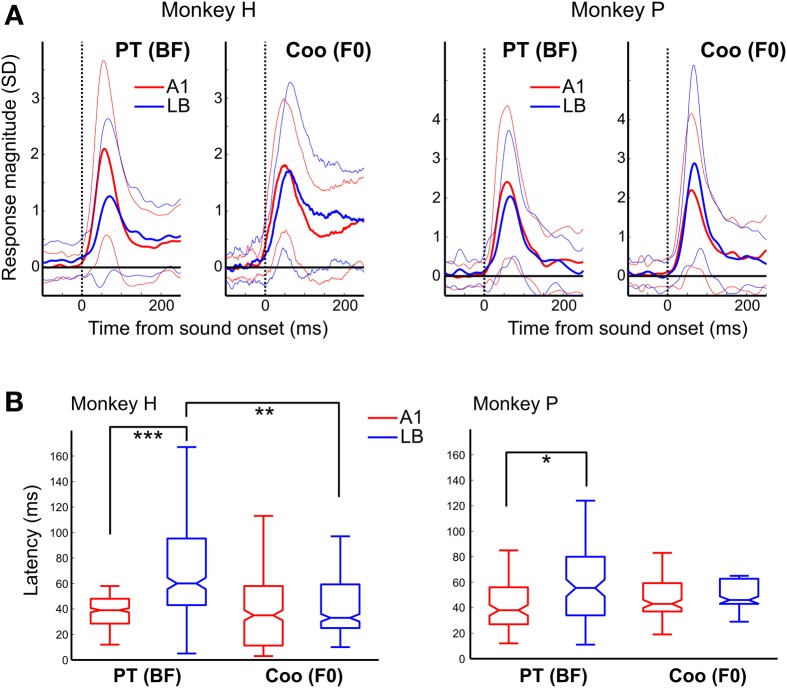
**Average response to the pure-tone BF and to coo calls with F0 matched to the BF**. **(A)** PSTH of the responses in A1 (red) and LB (blue) averaged separately across all auditory neurons to their BF (left panel) and to a coo (right panel) whose F0 was matched to the BF. The similarly color-coded thin lines show the standard deviations from the average response. The dotted vertical line indicates sound onset. Latencies to the BF in LB were longer than those to a coo (median ± SE, monkey H: median: 39 vs. 60 ms, Q1: 29 vs. 43 ms, Q3: 48 vs. 95 ms, *p* < 0.001; monkey P: median: 38 vs. 56 ms, Q1: 27 vs. 34 ms, Q3: 56 vs. 80 ms, *p* < 0.05). By contrast, there were no latency differences between A1 and LB in response to a coo (monkey H: median: 35 vs. 33 ms, Q1: 11 vs. 25 ms, Q3: 58 vs. 59 ms, *p* = 0.55; monkey P: median: 43 vs. 46 ms, Q1: 37 vs. 43 ms, Q3: 59 vs. 63 ms, *p* = 0.38). **(B)** Neural latencies to the BF in response to pure tones (PT) and to the coo with its F0 matched to the neuron's BF. Unlike the latencies observed in response to PTs and PNBs (Figure 3), there was no difference between A1 and LB latency in response to coo. Furthermore, the latency to coo was significantly shorter than the latency to the BF in LB at the population level (two animals: 43 ms vs. 59 ms, *p* < 0.01). The abbreviations used for the box and whisker plots are the same as in Figure 3. ^*^p < 0.05, ^**^p < 0.01, ^***^p < 0.001.

The gradual increase in minimum latency from A1 to ML, and from ML to AL in response to both PT and PNB (without pitch), as shown earlier in Figure 3C, suggests that this auditory information is processed hierarchically along these three subdivisions. Furthermore, the presence of harmonically-related interval ratios between peaks of multi-peaked neurons in their onset responses in LB but not in A1 (Figure 5C) suggests that harmonic processing occurs initially and preferentially in LB rather than A1. As one might predict from this hierarchy of PT processing (Figure 3C), the average BF latency was also longer in LB than in A1 (LB: median: 59 ms, *Q*1 = 42 ms, *Q*3 = 90 ms, *N* = 93; A1: 38 ms, *Q*1 = 27 ms, *Q*3 = 50 ms, *N* = 75; *p* < 10^−5^, Wilcoxon rank-sum test, Figure 7). By contrast, the latency to coos that were F0-matched to the BFs did not differ between LB and A1. This was due to the response latencies in LB to coos being significantly shorter than the response latencies to PTs at the BF (coo: median: 43 ms, *Q*1 = 28 ms, *Q*3 = 62 ms, *N* = 36; PT: 59 ms, *Q*1 = 42 ms, *Q*3 = 90 ms, *N* = 93; *p* < 0.01).

The above analysis restricts latency calculation to BF and the corresponding F0-matched coo. If minimum coo latency is calculated instead (i.e., the shortest latency of auditory responses elicited by all effective coos), similar results are obtained. Again, minimum coo latencies showed no difference between A1 and LB. In monkey H, the respective values were 40 vs. 43 ms (median, Q1:23 vs. 25 ms; Q3:57 vs. 61 ms, *p* = 0.80); in monkey P, they were 39 vs. 47 ms (median, Q1: 31 vs. 34 ms, Q3: 53 vs. 58 ms, *p* = 0.42). By contrast, the minimum response latency to PT increased significantly from A1 to LB (monkey H: median: 30 vs. 43 ms, Q1: 17 vs. 27 ms, Q3: 42 vs. 79 ms, *p* < 10^−4^; monkey P: median, 27 vs. 32 ms, Q1: 16 vs. 19 ms, Q3: 38 vs. 49 ms, *p* < 0.01). Corresponding latency data from single-unit recordings are displayed separately for the three subdivisions in Figure 3C.

## Discussion

We recorded single-unit activity from auditory core cortex (A1) and from the middle and anterior divisions of the lateral belt (LB) in response to pure tones and natural coo calls in two rhesus monkeys while they performed an auditory discrimination task. There were three major findings: (1) Latencies to pure-tone and pink-noise stimuli were significantly longer in LB than in A1; (2) responses to natural coo calls, which consist of complex harmonic tones with a defined fundamental frequency (F0), were observed with essentially equal latencies in LB and A1; together with finding 1, this suggests neuronal facilitation by communication calls with harmonic structures; and (3) although multi-peaked neurons were found in all three divisions, peak intervals in LB showed harmonic relationships in both early and late responses, whereas harmonic peak intervals in A1 were only found in modest numbers and only in late responses. These findings suggest that LB neurons play a critical role in the processing of auditory harmonics in animal communication calls.

### Latency differences between A1 and LB

The gradual increase of pure-tone latencies from A1 to LB (A1, 28 ms; ML, 35 ms; AL, 44 ms; Figure 3C) is comparable to that observed in other studies of macaques (Recanzone et al., 2000; Camalier et al., 2012). We observed a similar latency increase from **A1** to LB in response to pink noise bursts (PNB) (A1, 36 ms; ML, 39 ms; AL, 51 ms; Figure 3C). On the other hand, Lakatos et al. (2005) showed that the latency to a noise stimulus was reduced in belt regions compared to A1. However, their recording sites appeared to be in the posterior medial belt, whereas ours were in the lateral belt. More importantly, that study used narrow-band noise (NBN) stimuli, which elicit a pitch percept, as opposed to PNB stimuli that have no pitch. Thus, the reduced latencies to NBN in Lakatos' study correspond more closely to the relative latency reduction in response to (harmonic) coo stimuli reported here.

Since recent studies have reported that neuronal activity in the auditory cortex differ depending on task context or task demands (Scott et al., 2007; Sutter and Shamma, 2011; Niwa et al., 2012), neural latencies to S+ were analyzed separately for correct and incorrect trials. Although our results did not show a significant difference between the two conditions, it may be of interest to address this question more systematically in the future. This will require a more balanced design, since the number of error trials was very small (1.8%) in the present study.

Absolute latencies were longer overall in our study than in previous studies. One of the main reasons for this may be the use of raw data (in 1-ms bins) during the baseline period, which causes higher variability of baseline firing rates than does using Gaussian-smoothed data (see Methods). Furthermore, in our study, the variability of baseline firing rate across all stimuli was taken into account. Shorter latencies are generally observed in studies measuring multi-unit activity and current-source density responses, because neural latencies can be more clearly identified from such signals (Lakatos et al., 2005).

### Multi-peaked neurons and harmonic intervals

Multi-peaked neurons tuned to harmonically-related intervals have been reported in the primary auditory cortex of several species, including bats (Suga et al., 1979), marmosets (Kadia and Wang, 2003), and cats (Oonishi and Katsuki, 1965; Sutter and Schreiner, 1991; Eggermont, 2007; Noreña et al., 2008). Specifically, octave and perfect-fifth coding has been reported in A1 of cats (perfect fifth: Sutter and Schreiner, 1991) and marmosets (octave: Kadia and Wang, 2003). While all studies agree that spectral integration begins already at an early stage of auditory cortical processing, our study demonstrates that the number of neurons with harmonically-related intervals between best-frequency peaks increases significantly from A1 to LB (Figure 5). Furthermore, while we found multi-peaked neurons with harmonic intervals in both A1 and LB, there was a clear difference between the two regions in terms of response type: The distribution of peak distances in LB had a maximum at the perfect fifth for both early (<70 ms) and late response components (>70 ms) and a peak at one octave for late response components. By contrast, in A1 only a peak at the perfect fifth was found, and only for late response components (Figure 5C). Different (preferred) harmonic intervals were reported in A1 of cats (perfect fifth: Sutter and Schreiner, 1991), marmosets (octave: Kadia and Wang, 2003), and humans (Moerel et al., 2013) and it would be interesting to perform a cross-species comparison of preferred harmonic intervals in multipeaked responses as well as their cortical distribution in the future.

The relatively small amount of harmonic tuning observed in the early responses of A1 neurons suggests the possibility that LB is the first stage of convergence of inputs creating harmonic tuning, and that A1 neurons may reflect harmonic tuning mainly via feedback from higher-order regions like LB. Alternatively, it is possible that LB receives direct thalamic inputs that integrate over a broad frequency range at regular frequency intervals, or that inhibitory intracortical inputs play a role in sculpting the harmonically-related intervals. Taking all the evidence together, it seems most likely that convergent cortical projections from A1 create harmonically tuned cells in LB. This mechanism is commonly referred to as spectral “combination sensitivity” (Suga et al., 1979; Margoliash and Fortune, 1992; Rauschecker et al., 1995). The overall narrower tuning in A1 compared to LB observed in our study is consistent with this conclusion and is also supported by previous findings of others (Schroeder et al., 2001; Fu et al., 2004; Lakatos et al., 2005).

In one behavioral study, Izumi (2000) showed that Japanese macaques are poor at discriminating a single tone from simultaneously presented two-tone stimuli separated by either one octave or by a perfect fifth that share the same pitch. This suggests that the monkey makes use of perceptual grouping based on harmonically-related tones. Correspondingly, in another study (Kadia and Wang, 2003), response modulation was observed when sounds were presented outside the classical RFs of A1 in awake marmosets. Using a two-tone paradigm, these authors found that frequency-tuning peaks in multi-peaked neurons were often harmonically related, and they observed response facilitation when such harmonically related pairs of tones were presented simultaneously. Similar effects have been reported by other studies in A1 (Fitzpatrick et al., 1993; Brosch and Schreiner, 1997; Brosch et al., 1999; Kanwal et al., 1999), further supporting mechanisms of combination sensitivity. Since we did not employ a two-tone paradigm, direct response facilitation (increased firing rates) by a combination of tones was not examined here in either A1 or LB. Further studies will also be needed to examine whether neurons in A1 or LB are in fact more sensitive to consonant than to dissonant sound structures of a complex tone, since a recent study highlighted responses in primary auditory cortex to nonharmonic sounds (Fishman and Steinschneider, 2010).

### Responses to complex tonal “coo” calls

The average response to a PT at the best frequency (BF) and to a pitch-shifted coo at the same frequency also revealed that latencies to PTs were significantly shorter in A1 than in LB, whereas the response of LB neurons caused by adding higher harmonics to a fundamental frequency resulted in essentially equal latencies to natural coo calls in A1 and LB (Figure 7), a finding that may seem surprising given the standard view of hierarchical cortical processing. This finding further underscores that convergence of inputs in LB results in facilitation of responses to complex harmonic tones, as LB neurons generally prefer complex sounds over PTs (Rauschecker et al., 1995). Alternatively, responses to PTs and coos could depend on input from different divisions of the medial geniculate nucleus (MGN) with differential frequency tuning and latency (Hackett, 2011). Indeed, more multi-peaked neurons are found in the dorsal than in the ventral part of the MGN (Bartlett and Wang, 2011). We showed two possible neuron types that may contribute to the serial and parallel processing in A1 and LB (Figure 6 and Supplementary Figure 1). However, this classification was not able to cover all the neurons recorded in the PT and pitch-shifted coo paradigm because of the constraints on BF frequency ranges (see Methods). The relationships between the frequency tuning of the neurons and response latency (Figures 6, 7) remain unclear; specifically, we found only three neurons showing similar tuning to pitch-shifted coos and PTs (Supplementary Figure 1), and this needs to be addressed in further studies.

In sum, the findings of this study demonstrate that a purely serial model of cortical processing may be insufficient. On the other hand, the principles of hierarchical convergence and combination sensitivity in auditory processing (Rauschecker, 1998; DeWitt and Rauschecker, 2012) still stand. The latency reduction to harmonically-structured conspecific vocalizations and the existence of neurons tuned to simple frequency interval ratios in monkey nonprimary auditory cortex could be evidence of efficient information processing for ethologically relevant sounds. Harmonics are among the essential acoustic structures observed in natural acoustic environments that are generally limited to species-specific vocalizations (including human speech), which are the main sounds of biological interest for most species. In this study we employed a natural vocalization instead of synthetic stimuli to maximize our chances of eliciting neural responses, based on the evidence that neurons in the anterolateral belt area (AL) are more responsive to species-specific vocalizations (Tian et al., 2001). Since the previous study treated various harmonic and nonharmonic vocalizations as one category (“monkey calls”) and the F0 of the harmonic vocalizations was not varied, in this study we controlled the pitch and harmonic structure of monkey vocalizations by using a coo call, one of the most frequently heard vocalizations in both field and lab environments. Although the coo call has ethological meaning for the animals used in this study, we cannot determine from our results whether LB neurons respond to the harmonic structure of the calls, or whether they respond instead to complex acoustic features that might relate to their ecological relevance. Identification of the cortical areas that are involved in the transition from processing complex acoustic features (i.e., pitch and harmonicities) to processing natural conspecific calls is an important question. This issue is highlighted in a recent study by Fukushima et al. (2014) using microelectrocorticography in awake macaques: the classification of vocalizations was better than that for synthetic stimuli as the recording sites moved from caudal to rostral within the auditory ventral stream. Further studies will be needed to address this point at different neurological scales, including the single-unit level. Also, it would be of interest to learn more about the underlying neuronal mechanisms of harmonic preference observed at the behavioral level (Schellenberg and Trainor, 1996; Izumi, 2000) and when this important evolutionary development first occurred.

### Conflict of interest statement

The authors declare that the research was conducted in the absence of any commercial or financial relationships that could be construed as a potential conflict of interest.
