# Supplementary material for: Processing of harmonics in the lateral belt of macaque auditory cortex
Source: Front Neurosci. 2014 Jul 21;8:204. doi: 10.3389/fnins.2014.00204 (PMC4104551; doi:10.3389/fnins.2014.00204)
Supplement: Supplementary file 1 [file Presentation1.ZIP › 87319_Kikuchi_Data_Sheet_1.PDF]

Supplementary Materials for Kikuchi et al., 'Processing of harmonics in the lateral belt of macaque auditory cortex'

Yukiko Kikuchi[1,2,3*], Barry Horwitz[2], Mortimer Mishkin[3], Josef P. Rauschecker[1]

[1]Department of Neuroscience, Georgetown University Medical Center, Washington, DC, USA.

[2]Brain Imaging and Modeling Section, National Institute on Deafness and Other Communication Disorders, National Institutes of Health, Bethesda, MD, USA.

[3]Laboratory of Neuropsychology, National Institute of Mental Health, National Institutes of Health, Bethesda, MD, USA.

***Corresponding Author:** Dr. Yukiko Kikuchi, Institute of Neuroscience, Newcastle University Medical School, Newcastle upon Tyne, NE2 4HH, UK, yukiko.kikuchi@newcastle.ac.uk

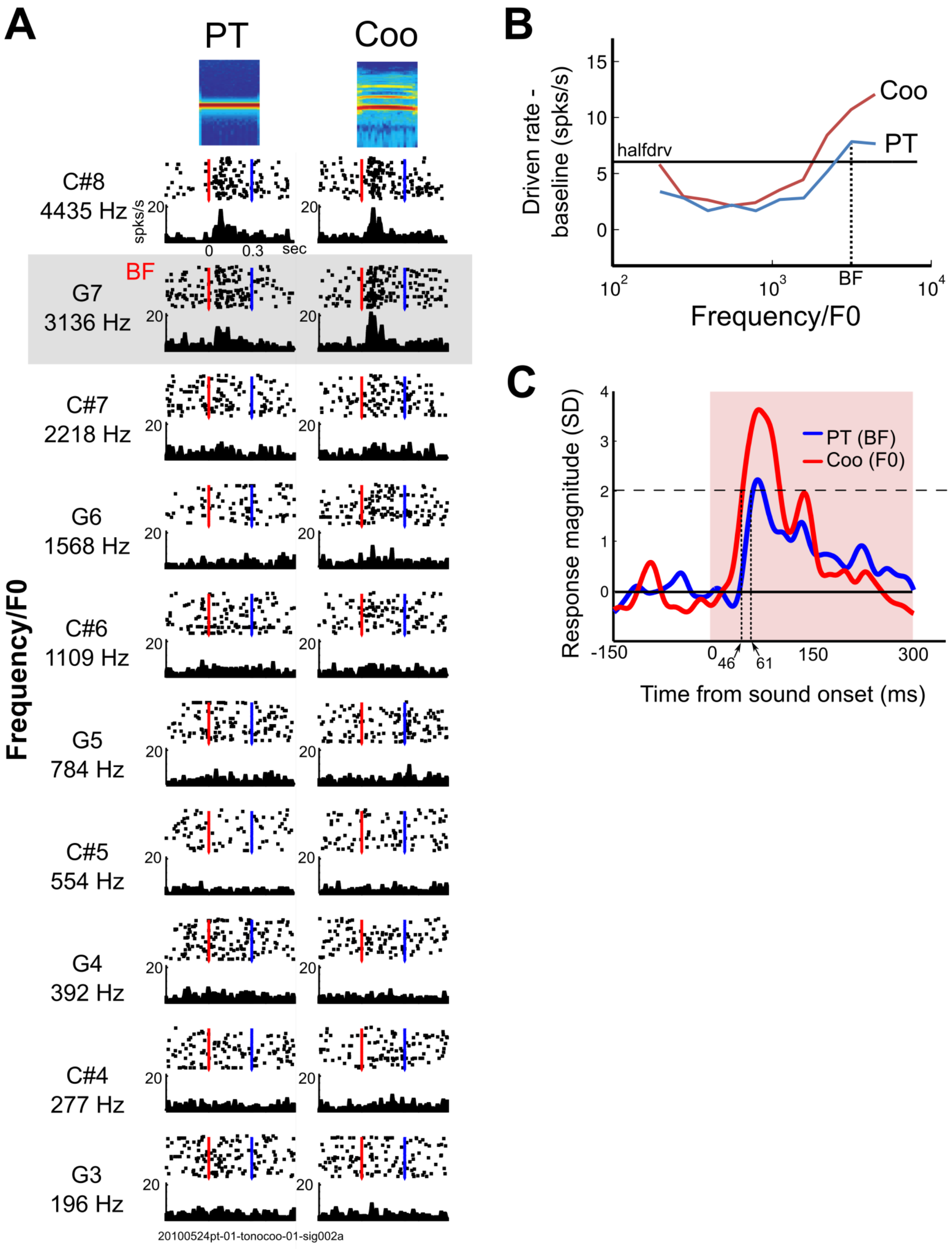

Supplementary Figure 1: Example of a neuron in LB showing similar tuning to pure tones and pitch-shifted coos. In some neurons, responses were elicited by a coo with the same pitch as the PT, even if the coo's overtones were outside the neuron's excitatory RF. (**A**) An example of an LB neuron (Unit B) that showed similar frequency tuning to PTs and pitch-shifted coos. (**B**) Tuning curves for Unit B shown in (**A**). This type of neuron responded at a high rate when the F0 fell within the neuron's RF, but not when the overtone harmonics fell within the same RF. (A and B). The BF for Unit B was 3136 Hz (G7). (**C**) Averaged PSTHs of the responses of Unit B to its BF (solid blue line) and to a coo (solid red line) whose F0 was matched to the BF. The onset latency was 61 ms compared to that to the pitch-matched coo of 46 ms, this neuron's minimum latency.

Supplementary Material 1: Pitch-shifted coo calls with F0 ranging from G3 (196 Hz) to C#8 (4435 Hz) in 6 semitone steps in a single audio clip.
